# Convergent Validity of Experimental Cognitive Tests in a Large Community Sample

**DOI:** 10.1177/10731911241283410

**Published:** 2024-11-10

**Authors:** Andy C. Dean, Jean-Baptiste Pochon, Robert M. Bilder, Fred W. Sabb, Eliza Congdon, Dara Ghahremani, Katherine H. Karlsgodt, Theo G. M. van Erp, Rebecca F. Schwarzlose, Tyrone D. Cannon, Nelson B. Freimer, Edythe D. London

**Affiliations:** 1 UCLA Semel Institute for Neuroscience and Human Behavior, Los Angeles, CA, USA; 2 UCLA Brain Research Institute, Los Angeles, CA, USA; 3 University of Oregon, Eugene, USA; 4 University of California, Irvine, USA; 5 Washington University School of Medicine in St. Louis, MO, USA; 6 Yale University, New Haven, CT, USA; 7 Department of Molecular and Medical Pharmacology, David Geffen School of Medicine, Los Angeles, CA, USA

**Keywords:** factor analysis, cognitive tests, neuropsychological, validity

## Abstract

Experimental cognitive tests are designed to measure particular cognitive domains, although evidence supporting test validity is often limited. The Consortium for Neuropsychiatric Phenomics test battery administered 23 experimental and traditional neuropsychological tests to a large sample of community volunteers (*n* = 1,059) and patients with psychiatric diagnoses (*n* = 137), providing a unique opportunity to examine convergent validity with factor analysis. Traditional tests included subtests from the Wechsler and Delis–Kaplan batteries, while experimental tests included the Attention Networks Test, Balloon Analogue Risk Task, Delay Discounting Task, Remember–Know, Reversal Learning Task, Scene Recognition, Spatial and Verbal Capacity and Manipulation Tasks, Stop-Signal Task, and Task Switching. Several experimental cognitive measures were insufficiently related to other tests and were excluded from factor analyses. In the remaining 18 tests, exploratory factor analysis and subsequent multigroup confirmatory factor analysis supported a three-factor structure broadly corresponding to domains of verbal/working memory, inhibitory control, and memory. In sum, several experimental measures of inhibitory control had weak relationships with all other tests, while the convergent validity of most tests of working memory and memory was supported.

Numerous cognitive tests have been designed and used in the fields of neuropsychology and cognitive neuroscience to assess specific constructs, although evidence of construct validity can vary widely between tests. Construct validity can be supported in several ways, including by evaluating whether test results predict expected outcomes based on theory (predictive validity), or whether the test relates to similar measures in an expected fashion (convergent validity). Convergent validity is often supported by factor analysis, in which several tests are administered to the same participants and it is determined whether the tests map onto a latent variable structure that would be expected based on theory.

The Consortium for Neuropsychiatric Phonemics (CNP) test battery presents a unique opportunity to evaluate the convergent validity of several experimental cognitive tests. CNP sought to identify behavioral endophenotypes and their genomic architecture as an approach to identifying tractable therapeutic targets that are common to complex neuropsychiatric syndromes (Poldrack et al., 2016). As part of this effort, 23 traditional and experimental cognitive tests, tapping the domains of cognitive control and memory, were administered to more than 1,000 community volunteers and smaller groups of individuals with schizophrenia (*n* = 52), bipolar disorder (*n* = 43), and attention-deficit hyperactivity disorder (ADHD; *n* = 42). DNA samples were taken for genomic analysis and self-report measures were obtained, and a subsample participated in magnetic resonance imaging. Prior reports on the CNP sample have been published (Bilder, Sabb, Cannon, et al., 2009; Bilder, Sabb, Parker, et al., 2009; Cao et al., 2021; Poldrack et al., 2016; Sabb et al., 2009).

Traditional cognitive tests have a long history of use in measuring cognitive function, although conceptualizations regarding their construct measurement may have changed over time. In contemporary iterations, most traditional cognitive tests have a test manual in which supportive evidence of validity is presented through factor analysis or correlational study. In CNP, traditional cognitive tests included subtests from the Wechsler Adult Intelligence Scale, fourth edition (WAIS-IV) and the Wechsler Memory Scale, fourth edition (WMS-IV), including Vocabulary, Matrix Reasoning, Digit Span, Letter–Number Sequencing, Symbol Span and Visual Reproduction, as well as the California Verbal Learning Test-II (CVLT-II), Stroop Task, Verbal Fluency, and Color Trailmaking Test. The test manual (see factor analyses in the WAIS-IV Technical and Interpretive Manual, 2008) supports the notion that Vocabulary and Matrix Reasoning tap the domains of verbal comprehension and perceptual reasoning, respectively, whereas Digit Span and Letter—Number Sequencing tap working memory (Wechsler et al., 2008; see also Bilder et al., 2023). Symbol Span and Visual Reproduction have high correlations with measures of visual memory and perceptual reasoning (Wechsler et al., 2009). Evidence in the test manual supports the view of the CVLT-II as a measure of verbal memory, with moderate positive correlations with measures of intelligence (Delis et al., 2000). The Stroop and Verbal Fluency tests in the Delis–Kaplan Executive Functioning System (D-KEFS) are positively correlated with measures of inhibitory control and language fluency (e.g., category fluency), respectively (Delis et al., 2001b). Finally, factor analyses in the manual for Color Trailmaking provide evidence that this measure taps divided attention and sequencing (D’Elia et al., 1996).

In comparison with traditional cognitive tests, experimental cognitive tests are typically developed outside of the clinical domain and are subjected to less empirical examination supporting validity. They are often developed with the intention of isolating a particular cognitive construct, potentially for use in functional neuroimaging applications (e.g., using the Stop-Signal Test to isolate the neural activity associated with inhibiting motor responses). Experimental cognitive tests in the CNP test battery were included with the intention of measuring aspects of response inhibition, working memory, and memory. Measures thought to be related to inhibition or impulse control included the Stop-Signal Task, Reversal Learning Task, Attention Networks Test, Task Switching, and the Delay Discounting Task, as well as a measure of risky decision-making, the Balloon Analogue Risk Task. Tests of working memory included the Spatial and Verbal Capacity Tasks and the Spatial and Verbal Maintenance and Manipulation Tasks, while those evaluating learning and memory included Remember–Know and Scene Recognition. Comprehensive evaluations have not been conducted to determine the relationship between these tasks and traditional measures of cognitive function, but some literature is available regarding correlates of task performance.

A typical summary variable from the Stop-Signal Task, stop-signal reaction time (SSRT), has been shown to have weak relationships with other self-report and performance measures of impulse control (Caswell et al., 2015; Gartner & Strobel, 2021; Sohn et al., 2014), although in one factor analytic study, it loaded on a factor of divided attention (Himi et al., 2021). Other research has found the Stop-Signal Task to be unrelated to verbal and nonverbal intelligence quotient (IQ) (Green et al., 2019). Likewise, factor analysis of the Balloon Analogue Risk Task has generally shown it to be unrelated to self-report measures of impulsivity and other performance-based measures of risky decision-making (Buelow & Blaine, 2015; Kim et al., 2018; Meda et al., 2009; Mejia et al., 2022). A typical metric of risk taking on the Balloon Analogue Risk Task, adjusted pumps, shows modest *positive* relationships with verbal IQ and visual learning, while it is not significantly correlated with some tests of executive function (Blair et al., 2018; Dean et al., 2011).

Different iterations of the Delay Discounting Task have been shown to be correlated with one another, but they are not typically related to performance-based measures of cognitive control including the Stop-Signal Task, Continuous Performance Task, or Go/No-Go Task (Caswell et al., 2015; MacKillop et al., 2016). The tendency to discount delayed rewards is negatively related to measures of intelligence (Dean et al., 2011; Shamosh et al., 2008).

Different variants of the Task-Switching paradigm have been shown to correlate with one another (constituting a “shifting” factor), and these also correlate with measures of executive function and overall cognitive ability (Himi et al., 2021). Reaction time on the “flanker” portion of the Attention Network Test is negatively related to measures of intelligence and processing speed in healthy volunteers (Troche et al., 2021). “Interference” scores on the flanker test (in which reaction time for congruent conditions is subtracted from incongruent conditions), however, show poor test–retest reliability (Jones et al., 2016). Errors on the reversal phase of the Reversal Learning Task are negatively correlated with estimated IQ and verbal working memory in individuals with schizophrenia, but are unrelated to measures of verbal learning and visual working memory (Waltz & Gold, 2007). Data on the relationship between other experimental measures administered in CNP and traditional cognitive measures are scant, particularly for tests of working memory and memory (e.g., Spatial and Verbal Capacity Tasks, Spatial and Verbal Maintenance and Manipulation Tasks, Remember–Know, and Scene Recognition).

The brief literature review cited above reveals that experimental cognitive tests that target specific domains may be related to some other measures of cognitive function, but it is unclear how these tests would relate to a broader battery of traditional cognitive tests. Furthermore, supportive evidence of convergent validity for several experimental tests of working memory and memory in the CNP battery has not been previously evaluated. This goal laid the groundwork for this study. We sought to examine how the experimental tests administered in CNP would relate to several traditional neuropsychological tests, and to one another.

Using one randomly selected half of the community volunteers in CNP (*n* = 529), we conducted an exploratory factor analysis (EFA) on all the cognitive tests included in the CNP study. Given the mixed findings previously reviewed, particularly for some measures of cognitive control, EFA was used so that model parameters could vary without restriction (i.e., as opposed to selecting a predefined factor structure). Following this EFA, we then conducted multigroup confirmatory factor analysis (MGCFA) to determine whether (a) the factor structure identified in the EFA was confirmed in the second half of the community sample (*n* = 530) and (b) whether the factors identified in the EFA appeared to be invariant to group membership (i.e., community volunteers or patients [*n* = 137]). In particular, research has shown that the factor structure underlying cognitive test performance can vary as a function of the participants included in the analysis (e.g., healthy subjects versus subjects with a memory disorder; Delis et al., 2003). We, therefore, sought to determine whether the test structure identified in the community volunteers also appeared to be appropriate in the psychiatric patients.

We hypothesized that traditional cognitive tests would relate to a factor structure that was generally consistent with that presented in their test manuals (see above). Regarding experimental tests, we hypothesized that purported measures of working memory (Spatial and Verbal Capacity Tasks, and Spatial and Verbal Maintenance and Manipulation Tasks) and Memory (Remember–Know and Scene Recognition) would factor together with traditional measures of these domains, respectively. Given the mixed findings previously discussed, we were uncertain how measures conjectured to assess cognitive control (e.g., Stop-Signal Task, task-switching, and Reversal Learning Task) would relate to traditional measures of these constructs, including the Stroop Task, Continuous Performance Task, and Color Trailmaking.

Finally, to gather additional evidence regarding external validity, we computed the effect size of the group difference between the community volunteers and patients on each cognitive test, with the intention of estimating the sensitivity of the tests to the types of cognitive differences that are present in psychiatric groups. Because meta-analytic data have shown that all three diagnostic groups (i.e., schizophrenia, bipolar disorder, and ADHD) exhibit medium to large effect-size deficits in working memory, memory, and executive function when compared to healthy control participants (Fatouros-Bergman et al., 2014; Pievsky & McGrath, 2018; Robinson et al., 2006), we hypothesized that the largest effect-size differences between the patients and community sample would occur on measures of working memory, memory, and cognitive control, with less clear expectations for group differences in risk taking and verbal abilities.

## Methods and Materials

We report how we determined our sample size, all data exclusions, all manipulations, and all measures in the study.

### Participants

The participants included 1,059 community volunteers and 137 patients. This included all participants collected in the CNP study who met the following inclusion and exclusion criteria. The patients consisted of individuals with Schizophrenia (*n* = 52), Bipolar I Disorder (*n* = 43), and ADHD (*n* = 42). Community volunteers were recruited from the Los Angeles metropolitan area using print advertisements, community postings/flyers, Internet postings (e.g., Craigslist), and presentations by investigators at selected community agencies (e.g., public library, community activity groups, and church groups). Patient groups were recruited through postings on patient-oriented websites, investigator presentations at patient-oriented group meetings at University of California, Los Angeles (UCLA) or in clinics of the Los Angeles County Department of Mental Health (LACDMH), and presentations to research teams and to participants in other ongoing studies at UCLA. All candidates were screened by telephone followed by informed consent and additional in-person screening to assure they met all study inclusion/exclusion criteria. All procedures were approved by the institutional review boards (IRBs) at UCLA and the LACDMH.

For both community and patient groups, participants were men or women ages 21 to 50 years; National Institutes of Health (NIH) racial/ethnic category either White, not Hispanic or Latino; or Hispanic or Latino, of any racial group; primary language either English or Spanish; adequate cooperation to complete assessments; completed at least 8 years of formal education; and visual acuity 20/60 or better. Exclusion criteria included significant medical illness (e.g., significant coronary disease, malignancy, immunodeficiency disorders, diabetes, cystic fibrosis, serious endocrine disorders, neurological or neuromuscular disorders, significant head trauma [loss of consciousness >30 minutes, repeat head injury; based on Head Injury Severity Questionnaire, available on request], stroke, seizures, neurosurgery, or blood dyscrasias) and testing positive for a drug of abuse [cocaine, methamphetamine, morphine, tetrahydrocannabinol (THC), and benzodiazepines] on urinalysis. Participants in the community group were excluded if they had lifetime diagnoses of schizophrenia or other psychotic disorder, Bipolar I or II disorder, current major depressive disorder (not in partial or full remission), suicidality, generalized anxiety disorder, or screening indicative of ADHD (greater than five positive symptoms for ADHD on the Adult ADHD Self-Report Scale v1.1, Part A; Kessler et al., 2005). Stable medications were permitted for the patient groups but exclusionary for the community group.

Diagnoses followed the *Diagnostic and Statistical Manual of Mental Disorders* (fourth ed., text rev.; *DSM-IV-TR*; American Psychiatric Association [APA], 2000) and were based on the Structured Clinical Interview for *Diagnostic and Statistical Manual of Mental Disorders* (fourth edition; *DSM-IV*; APA, 1994) (Structured Clinical Interview for *DSM*-IV Axis I Disorders [SCID-I]; First & Gibbon, 2004). In the patient groups, ADHD was assessed by interview with the Adult ADHD Clinical Diagnostic Scale (ACDS v1.2; Adler & Cohen, 2004). Interviewers/raters were trained to criteria as described elsewhere (Ventura et al., 1998). In brief, on a posttraining test, raters needed to show symptom-agreement overall kappa of 0.75, kappa specificity of 0.75, sensitivity of 0.75, and 0.85 kappa for diagnostic accuracy. Diagnostic and symptom elicitation skill was also assessed with the SCID Checklist of Interviewer Behaviors (Ventura et al., 1998) and the Symptom Checklist of Interviewer Behaviors (Ventura et al., 1993). Ongoing quality assurance checks documented kappa above .75 for each rater annually during the course of the study.

### Assessment Procedures

Based on the preferred language of participants, interviews and materials were provided in English or Spanish. Existing Spanish translations of English tests were used where available; otherwise, Spanish translations were completed by two investigators, Drs. Lidia Artiola-i-Fortuny and Xavier Cagigas. Participants who were bilingual (English and Spanish) were assessed for fluency in both languages using English and Spanish versions of the D-KEFS Verbal Fluency Test, with better performance determining the language of test administration.

Interviews, questionnaires, and neurocognitive assessments were typically completed in three sessions each lasting less than 4 hours. Community and patient groups received the same assessment battery except patients had additional clinical ratings of relevant psychopathology (e.g., questions regarding manic episodes in bipolar patients). Neurocognitive testing included both paper-and-pencil and computerized tests; two different counterbalanced orders of testing were used to control for order effects. A dual computer monitor system was employed for testing, with examiners “shadowing” participants and responding to questions or alerts as needed. E-Prime software (Psychology Software Tools, Inc.) was used for all computerized task programming, presentation, and response collection. Data were collected using a standard Dell keyboard on Dell workstations with Intel Core Duo processors, 2.4 or 2.2 GHz processing speed, 1 GB memory, and Dell 17” LCD monitors. The participants were positioned so that their eyes were centered approximately 20” from the monitor. All paper-and-pencil test results were double-entered into a secure online database, and computerized test results were automatically uploaded from testing computers to a secure server maintained by the CNP Data Management Unit.

### Cognitive Tests

#### Attention Network Test (Executive Section)

This is a computerized test of response inhibition based on the executive portion of the Fan et al. (2002) flanker task. On each of 144 trials, an arrow is presented in the center of the screen (above or below an initial fixation cross), surrounded on either side by two arrows or lines (“flankers”). On some trials, the flanker arrows point in the same direction as the central arrow (congruent condition—48 trials); on some, they point in the opposite direction (incongruent condition—48 trials); and on some, the flankers are lines (neutral condition—48 trials). The primary summary variable was the mean reaction time on incongruent trials.

#### The Balloon Analogue Risk Task

This is a computerized measure of risky decision-making (Lejuez et al., 2002). Balloons are presented on a computer screen, one per trial, and the examinee can “pump” the balloons up by pressing a response key to virtually inflate them. Each time a balloon is pumped up, five points are earned, and the objective is to earn as many points as possible. However, after a certain number of pumps (determined probabilistically), the balloon explodes, and no points are earned on that trial. On each trial, the participant can press a “cash out” key prior to the balloon explosion to retain points from the trial in a cumulative bank. In this version of the task, there are two conditions to assess performance in high- and low-risk contexts. Half of the balloons are colored blue (“low risk”—1/128 probability of explosion) and half red (“high risk”—1/32 probability of explosion); 20 of each color are presented in random sequence for a total of 40 trials. The primary summary variable was the total number of pumps on trials in which the balloon did not explode, referred to as “total adjusted pumps.” Adjusted pumps are counted instead of absolute pumps because explosions restrict the range of risk behavior (for evidence of the bias associated with absolute pumps, see Pleskac et al., 2008).

#### CVLT-II

This is a verbal list-learning task with 16 words that are read to the participant by the examiner on each of five learning trials (Delis et al., 2000). There are also measures of delayed recall, cued recall, and recognition memory. The total number of words recalled by the participant across the five learning trials was the summary variable of interest.

#### The Color Trailmaking Test

This is a paper-and-pencil version of the trail-making test that is designed to minimize the impact of language skills on test results (D’Elia et al., 1996). The task consists of two parts, A and B. Part A consists of a set of circles that are numbered from 1 to 25, colored either pink (even items) or yellow (odd items). The participant is tasked with drawing a line that connects the dots in order as quickly as possible. Part B presents 50 circles—yellow circles and pink circles—each numbered 1 to 25. The participant is required to quickly connect the circles from 1 to 25 but alternating between colors (pink and yellow). The primary summary variable was time to completion on part B.

#### Continuous Performance Test

This is a computerized “Go/No-Go” Continuous Performance Task similar to the Continuous Performance Test-II (Conners, 2000), in which letters are presented sequentially on a screen. The participant must respond with a button press, as quickly as possible, to every letter except the letter X. The interstimulus interval is variable and ranges from 750 to 3,750 ms. There are 360 trials, and 36 (10%) present the letter X. The primary summary variable to assess sustained attention was variability (standard deviation) in reaction time on “go” trials (note: Other possible summary variables, such as d′, hits, and commission errors, did not correlate well with other test scores; *r* < .30).

#### Delay Discounting Task

This is a measure of the tendency to discount the value of rewards as a function of the delay until receipt (Kirby et al., 1999). Although the task can be completed as a paper-and-pencil questionnaire, we administered the task on a computer, one trial at a time. Participants indicated their preference for one of two hypothetical options across 27 trials: One option consisted of receiving an amount of money immediately, and the other option consisted of receiving a larger amount of money at a later point in time. The discrepancy between the magnitude of reward (US$25 to US$85) and the duration of the delay period (7–186 days) is varied across trials. A hyperbolic statistical function can be fit to each trial to identify the point at which an individual is indifferent between the two options. Based on an individual’s preferences across the task, a summary discounting rate, or “total *k* value,” is derived (for details, see Kirby et al., 1999). Total *k* values were normalized with the natural log function in accordance with Kirby et al. (1999).

#### Remember–Know Task

This is a computerized test of episodic memory (based on the work by Tulving, 1985, see also van Erp et al., 2008 and Haut et al., 2015). On each of 60 encoding trials, participants are presented with stimulus pairs that consist of a target word (written in capital letters) and a colored picture of the word underneath it (e.g., the words say “WELL,” and there is red picture of a water well underneath it), combined with another word in lowercase and its associated picture (e.g., the word “man” with a drawing of the man underneath). Participants are encouraged to memorize as much about the items as possible, including the words, pictures, colors, and their location on the screen. The encoding phase is followed by a recognition phase, in which participants are presented with the 60 previously presented targets and 20 new foil words. Participants were instructed to press one key if they recalled the word and could remember associated stimuli (“remember”), another key if the word appeared familiar but could not recall additional details (“know”), or a third key if they thought the word was not previously shown (“unstudied”). Immediately following the recognition phase, participants completed a feature recognition phase in which they had to select (from three options) words that were paired with target words, or the color in which pictures were presented (they could also indicate that the stimulus was “unstudied”). The primary summary variable from the task was total accuracy across the recognition and feature recognition phases (both “remember” and “know” responses were considered correct for true positives), calculated as sensitivity (d′) based on the dual-process signal-detection theory (for details, see van Erp et al., 2008).

#### Reversal Learning Task

This is a computerized probabilistic Reversal Learning Task based on the work of Lawrence and colleagues (Lawrence et al., 1999). The task involves an initial training period in which participants must learn appropriate responses given probabilistic feedback (“noisy” feedback), followed by a reversal stage in which stimulus-response contingencies change and participants must re-learn new associations. On each trial, participants are presented with two round, abstract visual patterns (one on the left and one on the right) and asked to indicate which is “most likely to be correct” with the left and right keys, respectively. Feedback is provided to indicate whether a response is correct or incorrect (at first the participant is guessing). The feedback is probabilistic; some trials are entirely predictable (one stimulus is correct 100% of the time, and its pair is correct 0% of the time), whereas others are more unpredictable (i.e., 80% vs. 20%; 70% vs. 30%; and 60% vs. 40%). The learning phase continues for 160 trials, or until learning criteria are met (e.g., at least 70% accuracy on the entirely predictable pairs). After the learning phase, the reversal phase begins in which the correct response in half of the stimulus pairs is reversed (the 100% vs. 0% and 70% vs. 30% pairs), for a total of 40 trials. The primary summary variable was the proportion of trials that were correct during the reversal phase for the 100% vs. 0% items. In exploratory analyses, we also examined switching responses during the reversal phase on the 100% vs. 0% items after the first correct response, an index of perseveration/inconsistency (see Figure 2).

#### Scene Recognition Task

This is a computerized measure of recognition memory for photographs of outdoor scenes depicting either an urban or nature setting. In the encoding phase, participants were serially presented with 96 unique photographs, of which 40 were presented three times and 56 were presented once, resulting in 176 total trials. For each photograph, participants indicated with a key press whether the presented scene was an urban or natural setting. In the ensuing recognition phase, participants serially viewed 80 scenes that were composed of the 40 scenes presented thrice in the encoding phase, as well as 40 novel scenes. Participants indicated with a key press whether each scene was previously presented in the encoding phase (“old”) or not (“new”). The primary summary variable was total accuracy across the recognition phase as calculated through the d′ signal-detection metric.

#### Spatial Capacity Task

This is a computerized measure of spatial working memory based on the work of Glahn and colleagues (Glahn et al., 2002). On each trial, participants were shown a target array of one, three, five, or seven yellow circles positioned pseudo-randomly around a fixation point for 2 seconds. After a delay of 3 seconds, a single green circle appeared, and participants were required to indicate whether that circle was in the same position as any one of the previously shown yellow circles. There were 48 trials, 12 for each memory load (1, 3, 5, or 7). The primary summary variable was accuracy across all trials as calculated through the d′ signal-detection metric.

#### Spatial Maintenance and Manipulation Task

This is a computerized measure of spatial working memory based on the work of Cannon and colleagues (Cannon et al., 2005). On each trial, participants were shown a target array of three yellow circles positioned pseudo-randomly around a fixation point for 1.5 seconds, followed by a prompt to “hold” or “flip” the information. “Hold” indicated that the participant should remember the spatial location of the three dots, while “flip” indicated that they should remember the spatial location of the three dots if their orientation was “flipped” along the horizontal axis (i.e., dots below the axis would be positioned, respectively, above the axis, and vice versa). After this prompt, three green dots were presented and the participant was tasked with indicating if the green dots were in the correct place depending on the “hold” or “flip” instructions, respectively (yes or no). There were 20 trials of each type (40 in all), presented in random order. The primary summary variable was response accuracy for “flip” trials calculated through the d′ signal-detection metric.

#### Stop-Signal Task

This is a computerized measure of motor response inhibition (Logan, 1994; Logan & Cowan, 1984). On each trial, an “X” or an “O” appeared as a “go” stimulus in the center of the screen, and participants were told to press the left arrow button when they saw an “X” and the right arrow button when they saw an “O.” On a subset of trials (25%), a stop signal (a 500-Hz tone) was presented a short delay after the “go” stimulus (stop trials). Participants were instructed to respond as quickly and accurately as possible on all trials, but to withhold their response on stop trials. On stop trials, the delay of the onset of the stop signal, or stop-signal delay, was varied, such that it was increased after the participant successfully inhibited their response to a stop signal (making the next stop trial more difficult) and decreased after the participant failed to inhibit their response to a stop signal (making the next stop trial less difficult). This one-up/one-down tracking procedure ensured that subjects successfully inhibited on approximately 50% of inhibition trials. The primary summary variable was the SSRT, an estimate of the time required for the participant to stop their responding, calculated according to the “quintile” method (see Band et al., 2003; Congdon et al., 2012). Unacceptable SSRTs were excluded from analysis in accordance with criteria set forth by Congdon et al. (2012).

#### Stroop Color-Word Test

This is a classic test of inhibitory control; in this study, it was administered by computer (see Holmes & Pizzagalli, 2008). On each trial, one of three color words were presented (red, green, or blue); these words were either printed in congruous color (e.g., “red” printed in red text) or incongruous color (e.g., “red” printed in blue text). The participant was instructed to quickly press one of three keys corresponding to the color of text the word was printed in, not what the word stated. Forty-two practice trials were administered to familiarize participants with the task and to reinforce mapping of the keys to the appropriate responses. In the experimental phase, 152 trials were administered, 54 of which were incongruent (36%). The primary summary variable was the mean response time on incongruent trials in the experimental phase.

#### Task Switching

This is a computerized measure of the ability to change mental set based on task demands and based on the work of Monsell (2003). Stimuli involved one of two shapes (triangle or circle) represented in one of two colors (green or red). On each trial, the participant needed to indicate, with a button press, either the shape of the stimulus (triangle or circle) or the color it was presented in (green or red), as quickly as possible (one button was used for triangle or red and another for circle and green; button mapping configurations were counterbalanced across participants). Immediately prior to each trial, participants were provided a cue indicating which aspect of the stimulus they should respond to, either shape (shape or “S”) or color (color or “C”). After practice trials, participants completed 192 trials, 64 of which (33%) involved a switch in task demands from the previous trial (i.e., required responding to shape instead of the color, or vice versa). The primary summary variable was the mean reaction time on trials that involved a switch in task demands from the preceding trial.

#### Verbal Capacity Task

This is a computerized measure of working memory that was created to be the verbal analog to the Spatial Capacity Task (see Karlsgodt et al., 2007). On each trial, a target set of three, five, seven, or nine yellow uppercase consonant letters was displayed for 2 seconds, followed by a 3-second fixation. A green lowercase letter then appeared for 3 seconds, and subjects indicated whether the green letter matched any of the letters in the target set (yes or no). There were 48 trials, 12 for each memory load (1, 3, 5, or 7). The primary summary variable was accuracy across all trials as calculated through the d′ signal-detection metric.

#### Verbal Fluency

This is a classic test of the ability of participants to generate words beginning with a certain letter (F, A, and S), taken from the D-KEFS (Delis et al., 2001a). Participants were provided 1 minute for each letter. The summary variable was the total number of words generated across all three letters.

#### Verbal Maintenance and Manipulation Task

This is a computerized measure of working memory created to be the verbal analog to the Spatial Maintenance and Manipulation Task, based on the work of Kim and colleagues (Kim et al., 2004). On each trial, participants were presented with four sets of two-letter syllables (e.g., Ro, Bi, La, and Te), ordered from left to right, for 1.5 seconds, followed by a prompt to “hold” or “order” the information. “Hold” indicated that they should remember the spatial location of the syllables, while “order” indicated that they should mentally alphabetize them from left to right. After this prompt, a single syllable was presented (e.g., Ro) in one of four placeholder locations ordered from left to right (denoted by lines), and the participant was tasked with indicating if the syllable was in the correct place depending on the “hold” or “order” instructions, respectively (yes or no). There were 20 trials of each type (40 total), presented in random order. The primary summary variable was the response accuracy for “order” trials calculated through the d′ signal-detection metric.

#### WAIS-IV: Vocabulary, Matrix Reasoning, Digit Span, and Letter–Number Sequencing

Traditional tests included subtests from the WAIS-IV (Wechsler et al., 2008). The Vocabulary subtest examines the ability to define words, while Matrix Reasoning measures the ability to solve visual puzzles; the two subtests together are widely used to provide an estimate of general intellectual ability (Wechsler et al., 2008). Digit Span is a measure of working memory in which strings of numbers are read aloud, and the participant recites them forwards, backward, and in numerical order. Letter–Number Sequencing is a measure of working memory in which numbers and letters are read aloud and the participant needs to recite them in numerical and alphabetical order. Summary variables consisted of total raw scores for each subtest.

#### WMS-IV: Symbol Span and Visual Reproduction

Traditional tests included subtests from the WMS-IV (Wechsler et al., 2009). Symbol Span is the visual analogue to the forward Digit Span subtest, in which strings of symbols are briefly shown to the participant, and the participant needs to recall them in their proper sequence (in a recognition format by discriminating them from foils). Visual reproduction is a test of visual memory in which abstract line drawings are briefly shown to the participant, who must draw them from memory with paper-and-pencil, both immediately and after a 20 to 30 minutes delay. Summary variables consisted of the total raw score for symbol Span and total delayed recall (aka Visual Reproduction II) for visual reproduction.

### Data Analysis

To reduce the risk of deriving task-based factors (Cattell, 1961; Larrabee, 2003), one summary variable was identified from each cognitive test for implementation in factor analyses. This consisted of the variable felt to be most representative of the construct targeted by each task (see task descriptions). Other possible summary variables were examined in exploratory analyses for some tasks (see “Preliminary Analyses” section). All analyses were conducted in the R statistical package (3.6.0) using *lavaan* (0.6–8), psych (2.0.12), and semPlot (1.1.2) libraries. Differences between the community and the patient groups were assessed with *t*-tests or chi-square tests, as appropriate. Suitability of the data for factor analysis was first determined by examination of intervariable correlations, Bartlett’s test of sphericity, Kaiser–Meyer–Olkin measure of sampling adequacy, the anti-image correlation matrix, and the determinant of the correlation matrix. The community sample was randomly divided into two halves. EFA was conducted on the first half of the community sample (*n* = 529), while MGCFA was used to test invariance of the factors identified in the EFA in the second half of the community sample (*n* = 530) and patients (*n* = 137). EFA was conducted using principal axis factoring (assuming the presence of some measurement error) with direct oblimin rotation (allowing for known correlations between different cognitive functions) using the “fa” function from the *psych* package. Missing cases were excluded on a pairwise basis given some variability in the sample size of each test (most tests had 15 or fewer missing values but the Remember–Know test had 203 missing values; a temporary computer programming error resulted in missing data for this test). Parallel analysis with Monte Carlo simulation was used to help determine the number of factors to retain in the EFA. MGCFA tested the invariance of the model derived through the EFA by conducting CFAs and successively adding constraints on the equality of factor loadings and item thresholds across participant groups (see “Results” section). All MGCFA analyses were performed using “cfa” function from the *lavaan* package. Model fit was determined using the chi-square likelihood test, root mean square error of approximation (RMSEA < .08 acceptable, < .05 good fit; MacCallum et al., 1996; van de Schoot et al., 2012), standardized root mean square residual (SRMR < 0.08 good fit; Hu & Bentler, 1999), and comparative fit index and Tucker–Lewis index (CFI and TLI > .90 acceptable, > .95 good fit; Byrne, 2012). ΔCFI (i.e., invariance if > −0.10; Chen, 2007) was used as an additional marker of model comparisons. Finally, analysis of covariance (ANCOVA) was used to determine the group difference effect size on each cognitive test (and factor scores derived from factor analyses), while controlling for age and gender.

## Results

### Preliminary Analyses

The two randomly selected halves of the community sample did not differ significantly in age, years of school attended, Vocabulary score on the WAIS-IV, gender, ethnicity, race, or primary language (Spanish or English) (all *p* > .05). In contrast, compared with the community samples, the patient group had lower Vocabulary scores, more Spanish speakers, more males, and less education (all *p* < .01). The patients also had a lower proportion of Native American individuals, but a greater proportion of individuals of more than one race (*p* < .01; see Table 1).

**Table 1:** Characteristics of Research Participants

Characteristic	Community Group 1	Community Group 2	Patient group
Sample size	529	530	137
Age	31.3 ± 8.6	31.7 ± 8.4	34.1 ± 9.4**
Education (years)	15.0 ± 2.0	15.1 ± 2.0	13.8 ± 2.1**
Vocabulary score^a^	12.0 ± 3.1	12.1 ± 3.3	11.1 ± 3.8**
Gender (male/female)	246/283	237/293	85/52**
Ethnicity (Hispanic/Non-Hispanic)	229/300	201/329	52/85
Primary language (English/Spanish)	452/77	457/73	132/5**
Race			
White	375	410	101
Black/African American	5	5	4
Asian	2	2	2
Hawaiian/pacific islander	1	0	0
American Indian/Alaska native^b^	131	100	17**
More than one race	10	9	11**

*Note*. Values are *M*±*SD*s. The symbol ** indicates a significant difference from the control groups at *p* < .01.

a Age-corrected scaled score from the Vocabulary subtest of the WAIS-IV. ^b^ Note that many Hispanic participants identified Native American Indian heritage.

Correlations between the cognitive test scores were evaluated in the entire sample to determine suitability for factor analysis (see Table 2). The Delay Discounting Task, Reversal Learning Task, Spatial Maintenance and Manipulation Task, Balloon Analogue Risk Task, and Stop-Signal Task had low correlations with all other test scores (all *r* < .28). In a preliminary EFA (retaining all factors with eigenvalues >1, resulting in a five-factor model), these tests also had very low communalities (communalities < 0.18), indicating that they shared little variance with all factors derived from the model. Measures with communalities <0.20 are typically considered inappropriate for factor analytic techniques (Child, 2006). These cognitive tests were, therefore, excluded from subsequent factor analysis.

**Table 2:** Pearson Correlations Between Cognitive Tests in the Entire Sample (n = 1,196)

Test	ANT	BART	CPT	CVLT	DDT	DS	LNS	MR	RK	RLT	SCAP	SMNM	SP	SR	SST	Stroop	Trails	TS	VCAP	VF	VMNM	Voc	VR
ANT	—																						
BART	−.18	—																					
CPT	.33	−.02	—																				
CVLT	−.20	.11	−.20	—																			
DDT	.10	−.11	.01	−.13	—																		
DS	−.34	.25	−.20	.29	−.16	—																	
LNS	−.29	.22	−.16	.33	−.14	.71	—																
MR	−.42	.24	−.17	.38	−.21	.52	.46	—															
RK	−.15	.06	−.19	.36	−.12	.23	.22	.28	—														
RLT	−.06	.04	−.10	.04	−.04	.08	.07	.10	.06	—													
SCAP	−.23	.12	−.26	.25	−.09	.30	.26	.30	.25	.05	—												
SMNM	−.23	.09	−.14	.16	−.06	.27	.25	.28	.13	.03	.24	—											
SP	−.36	.22	−.20	.43	−.15	.48	.49	.52	.34	.08	.31	.26	—										
SR	−.16	.14	−.18	.26	−.05	.20	.21	.31	.25	.03	.25	.14	.36	—									
SST	.24	−.05	.24	−.19	−.05	−.16	−.16	−.14	−.12	−.00	−.16	−.07	−.20	−.14	—								
Stroop	.48	−.10	.31	−.22	.08	−.27	−.24	−.27	−.16	−.05	−.19	−.16	−.27	−.10	.17	—							
Trails	.43	−.17	.26	−.34	.16	−.46	−.43	−.48	−.21	−.05	−.32	−.25	−.48	−.22	.18	.39	—						
TS	.52	−.16	.35	−.32	.06	−.39	−.35	−.38	−.21	−.10	−.24	−.19	−.39	−.23	.26	.46	.48	—					
VCAP	−.20	.14	−.14	.25	−.10	.44	.38	.33	.21	.06	.30	.13	.29	.22	−.15	−.15	−.29	−.26	—				
VF	−.27	.19	−.13	.23	−.17	.48	.47	.40	.22	.08	.17	.18	.36	.19	−.14	−.18	−.39	−.31	.34	—			
VMNM	−.23	.14	−.16	.30	−.17	.52	.49	.43	.21	.06	.29	.24	.43	.21	−.13	−.18	−.42	−.29	.40	.41	—		
Voc	−.23	.28	−.10	.29	−.25	.49	.45	.46	.18	.06	.20	.17	.37	.24	−.09	−.09	−.37	−.26	.26	.50	.38	—	
VR	−.32	.16	−.17	.45	−.14	.29	.34	.42	.24	.07	.31	.21	.52	.34	−.10	−.27	−.40	−.35	.25	.25	.28	.26	—

*Note.* ANT = Attention Network Task (executive section); BART = Balloon Analogue Risk Task; trails = Color Trailmaking Part B; CPT = Continuous Performance Task Reaction Time Variability; CVLT = California Verbal Learning Test-2 Total Learning; DDT = Delay Discounting Task; DS = Digit Span (WAIS-IV); LNS = Letter–Number Sequencing (WAIS-IV); MR = Matrix Reasoning (WAIS-IV); RK = Remember–Know; RLT = Reversal Learning Task; SR = Scene Recognition; SCAP = Spatial Capacity Task; SMNM = Spatial Maintenance and Manipulation; SST = Stop-Signal Task; Stroop = Stroop Color-Word; SP = Symbol Span (WMS-IV); TS = Task Switching; VCAP = Verbal Capacity Task; VF = Verbal Fluency; VMNM = Verbal Maintenance and Manipulation; VR = Visual Reproduction II (WMS-IV); Voc = Vocabulary (WAIS-IV).

As described in the cognitive tests section, the primary summary variable for each test was that considered most conceptually representative of each task (see task descriptions). For exploratory purposes, we also examined alternative summary variables for the tests that had low correlations with all other tests. This included alternative summary variables for the Delay Discounting Task (e.g., nonlog total *k* value and *k* value for different reward magnitudes); Balloon Analogue Risk Task (e.g., number of explosions, standard deviation of pumps, coefficient of variation of pumps, and pumping on balloons of specific risk probability); reversal learning (number correct on the reversal phase for probabilistic items, restricting analyses to only participants with good performance on the learning phase, errors during the reversal phase, overall selection of high probability items, switching responses during the reversal phase, etc.); stop signal (e.g., nonquant SSRT, stop-signal delay, SSRT on the first or second block of trials); and spatial maintenance and manipulation (e.g., manipulation trials total accuracy, maintenance trials d’, maintenance trials total accuracy, hits, and false alarms). None of these alternative variables had higher correlations with other tests than the primary summary variables.

It should also be noted that although the Stroop Task, Attention Network Test, and Task Switching allowed for the calculation of interference scores (i.e., scores in which the reaction time for congruent stimuli was subtracted from the reaction time for incongruent stimuli), these interference scores had low correlations with all other tests, including other interference tests (all *r* < .20). The unadulterated mean reaction times from the incongruent conditions, however, had higher correlations with other tests (*r* > .35).

Preliminary EFA revealed acceptable values on Bartlett’s test of sphericity (*p* < .001), Kaiser–Meyer–Olkin Measure of Sampling Adequacy (0.923), anti-image correlation matrix (diagonals all > 0.89), and the determinant of the correlation matrix (determinant = 0.001). The data were, therefore, deemed to be appropriate for factor analysis.

### EFA in First Half of Community Sample (n = 529)

With respect to EFA in the first half of the community volunteers, parallel analysis based on eigenvalues derived from principal components analysis suggested the presence of three nontrivial factors (i.e., three factors with eigenvalues exceeding that produced by random data). To further explore the appropriate factor solution, EFAs were run on models containing between one and eight factors and fit indices were examined.

Among the different models, Bayesian information criteria (BIC) indicated that a three-factor model afforded the best fit (i.e., had the lowest value; Bollen et al., 2014). RMSEA likewise met criteria for “good fit” (MacCallum et al., 1996; van de Schoot et al., 2012) with a three-factor solution (RMSEA = 0.052). TLI found a three-factor model to be acceptable (TLI = 0.925; see Byrne, 2012), but a four-factor model to be slightly better (TLI = 0.949; see Byrne, 2012). When the pattern matrix was compared between a three- and a four-factor model, the three-factor model provided the most clear-cut pattern of factor loadings; in contrast, the four-factor model had only two tests loading on the fourth factor, with one of these tests loading equally on more than one factor. In review of the above findings, a three-factor solution was determined to provide the most parsimonious fit, with additional confirmation to be determined by MGCFA.

The pattern matrix for the three-factor solution is provided in Table 3. The first factor comprised largely measures of working memory and verbal abilities, with the highest loading observed for Digit Span (Digit Span, Letter–Number Sequencing, Verbal Fluency, Vocabulary, Verbal Maintenance and Manipulation Task, Verbal Capacity Task, Matrix Reasoning, and Color Trailmaking; listed from highest to lowest in loading). The second factor comprised tests of timed inhibitory control, with the highest loading produced by the Attention Network Test (Attention Network Test, Stroop Task, Task Switching, Continuous Performance Task, and a moderate loading with Color Trailmaking). The third factor comprised primarily tests of memory, with the highest loading produced by Visual Reproduction II (Visual Reproduction II, Symbol Span, CVLT-II, Remember–Know, Scene Recognition, Spatial Capacity Task, and Matrix Reasoning).

**Table 3:** Pattern Matrix From EFA on First Half of Community Subjects (N = 529)

Test	Factor 1	Factor 2	Factor 3
Digit span	**.911**	−.040	−.150
Letter–Number Sequencing	**.772**	.003	−.031
Verbal Fluency	**.598**	−.085	−.022
Vocabulary	**.556**	.069	.110
Verbal Maintenance and	**.522**	−.008	.157
Manipulation			
Verbal Capacity Task	**.479**	.002	.091
Matrix Reasoning^a^	**.378**	−.139	.317
Color Trailmaking Part B^a^	−**.356**	.276	−.172
Attention Network Task (executive section)	−.061	**.757**	.074
Stroop Color-Word	.034	**.634**	.026
Task Switching	−.139	**.587**	−.046
CPT reaction time	.031	**.377**	−.111
Variability			
Visual Reproduction II	−.086	−.132	**.650**
Symbol Span	.154	−.070	**.616**
CVLT-II Total Learning	.054	.013	**.533**
Remember–Know	.023	.067	**.478**
Scene Recognition	−.006	.005	**.465**
Spatial Capacity Task	.036	−.053	**.347**

*Note.* Factor loadings shown in bold reflect the highest loading for that test; the tests are considered to “load” on that factor. ^a^ Note that some cross loading was observed for Color Trailmaking Part B (Factors 1 and 2) and matrix reasoning (Factors 1 and 3).

### MGCFA

**Figure 1:**
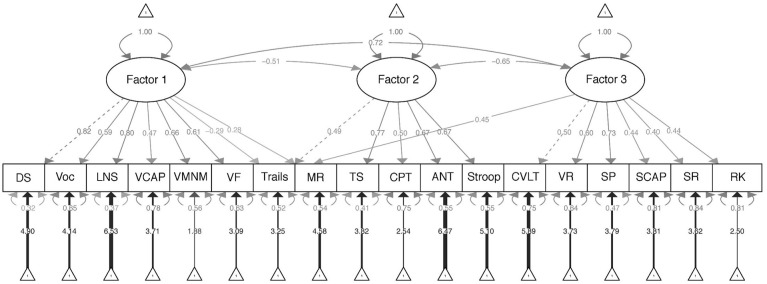
Three-Factor Model Based on Confirmatory Factor Analysis of Tests Included in the CNP Battery *Note.* ANT = Attention Network Task (executive section); BART = Balloon Analogue Risk Task; trails = Color Trailmaking Part B; CPT = Continuous Performance Task Reaction Time Variability; CVLT = California Verbal Learning Test-2 Total Learning; DDT = Delay Discounting Task; DS = Digit Span (WAIS-IV); LNS = Letter–Number Sequencing (WAIS-IV); MR = Matrix Reasoning (WAIS-IV); RK = Remember–Know; RLT = Reversal Learning Task; SR = Scene Recognition; SCAP = Spatial Capacity Task; SMNM = Spatial Maintenance and Manipulation; SST = Stop-Signal Task; Stroop = Stroop Color-Word; SP = Symbol Span (WMS-IV); TS = Task Switching; VCAP = Verbal Capacity Task; VF = Verbal Fluency; VMNM = Verbal Maintenance and Manipulation; VR = Visual Reproduction II (WMS-IV); Voc = Vocabulary (WAIS-IV).

A three-factor CFA was run based on the results from the EFA previously described, with each test loading on their identified factor (with the exception that Color Trailmaking and Matrix Reasoning were each allowed to load on two factors, respectively, given cross loadings [dual loadings > 0.25] observed in the EFA; see Table 3). As shown in Table 4, fit indices from this CFA conducted in Community Group 2 indicated that the three-factor model was a good fit to the data (RMSEA = 0.05; CFI = 0.94; TLI = 0.93; SRMR = 0.04). The fit statistics for the CFA in the patient group were inferior to those in the community group but were still generally within acceptable ranges (RMSEA = .073; CFI and TFI > 0.90; SRMR = .057), especially considering the small size of the patient group. Expectedly, fit statistics for the combined sample of Community Group 2 and patients were also acceptable (RMSEA = .05; CFI = 0.94; TFI = 0.93; SRMR = 0.04). As shown in Figure 1, factor loadings for the cognitive tests in the CFA (conducted in Community Group 2) were generally in agreement with those obtained in the original EFA.

**Table 4:** Model Fit Indices and Model Comparison Statistics for Measurement Invariance Models Across Community and Patient Groups

Sample/analysis	RMSEA	CFI	TLI	SRMR	Chi-square	DF	ΔCFI
Community Group 1 EFA (*n* = 529)	.052	—	.92	.04	—	102	—
Community Group 2 CFA (*n* = 530)	.050	.94	.93	.041	301.6	130	—
Patient group CFA (*n* = 137)	.073	.91	.90	.057	226.2	130	—
Community Group 2 + patients CFA (*n* = 667)	.054	.94	.93	.039	380.9	130	—
Configural invariance model	.055	.93	.92	.044	527.8	260	−.007
Weak invariance model	.055	.93	.92	.052	561.3	277	−.004
Scalar invariance model	.056	.92	.92	.055	595.1	292	−.005
Invariance uniqueness model	.070	.88	.88	.070	813.1	310	−.050

MGCFA was run across the community (Group 2) and patient groups. The *configural invariance* model set no equality constraints across groups for any model parameter estimates. This model had adequate fit (RMSEA = 0.055; CFI and TFI > 0.92; SRMR = 0.044), indicating that the three-factor model fit both groups independently (see Table 4).

Compared with the *configural invariance* model, the *weak invariance* model added the constraint that factor loadings needed to be equivalent between groups. The *weak invariance* model was a good fit to the data (RMSEA = 0.055; CFI and TFI > 0.92; SRMR = 0.052) and showed no significant worsening in fit compared with the *configural model* (Δ CFI > −0.01; see Chen, 2007). This finding suggested that the relationship between performance on the cognitive tests and the constructs measured (i.e., factors) could be considered equivalent between the community and patient groups.

Compared with the *weak invariance* model, the *scalar invariance* model added the constraint that the intercepts for the cognitive tests were equivalent across groups. This model also showed adequate fit (RMSEA = 0.056; CFI and TFI > 0.92; SRMR = 0.055), with no significant worsening in fit compared with the *weak invariance* model (Δ CFI > −0.01). This finding suggested that the magnitude of scores on the cognitive tests had the same effect on factor scores irrespective of group membership (i.e., test scaling was equivalent between groups).

Finally, compared with the *scalar invariance* model, the *invariance uniqueness* model added the constraint that error variances needed to be equivalent between groups. Fit indices from the invariance uniqueness model were variously below recommended cutoffs (RMSEA = 0.070; CFI = 0.88; SRMR = 0.070); this model also significantly changed fit compared with the *scalar invariance* model (Δ CFI < −0.01). This suggested possible group differences in reliabilities of the cognitive test scores, or the presence of unmodeled extraneous variables that had differential effects in the different groups (see Wu et al., 2007).

Across cognitive tests, we investigated which residual coefficients (i.e., error variance) differed the most across groups and iteratively relaxed them (i.e., allowed them to differ freely between groups) to test for partial invariance. Doing so incrementally decreased the delta CFI, but it never reached the well-accepted cutoff of delta CFI > −0.01, indicating that partial invariance was not met. The small sample size of the patient group (*n* = 137) likely contributed to these findings.

### Group Difference Effect Sizes

**Figure 2.**
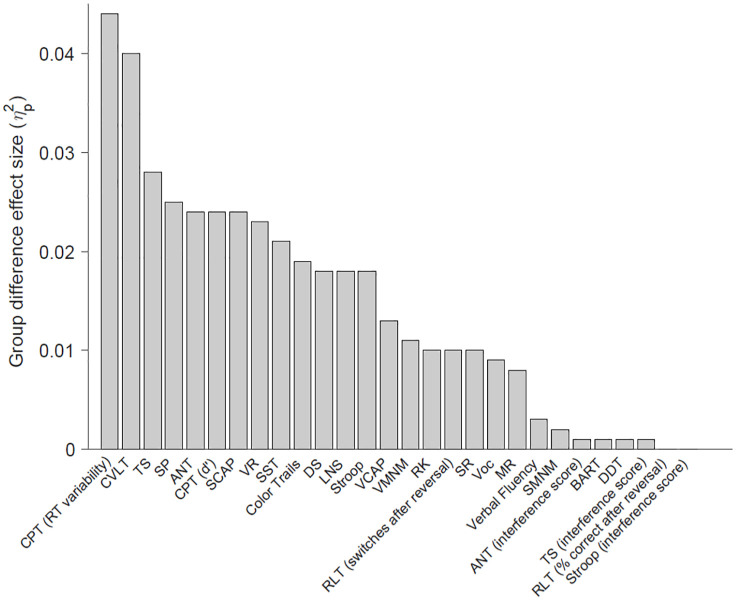
Effect Size of the Difference Between Community Volunteers (n = 1,059) and Patients (n = 137) on Tests Included in the CNP Battery. *Note.* Group difference effect size (η_p_^2^), after controlling for age and gender. ANT = Attention Network Task (mean reaction time on inhibitory trials); ANT (interference score) = Attention Network Task interference (inhibitory reaction time—congruent reaction time); BART = Balloon Analogue Risk Task; Color Trails = Color Trailmaking Part B; CPT (RT variability) = Continuous Performance Task Reaction Time Variability; CPT (d’) = Continuous Performance Task d-prime; CVLT = California Verbal Learning Test-2 Total Learning; DDT = Delay Discounting Task; DS = Digit Span (WAIS-IV); LNS = Letter–Number Sequencing (WAIS-IV); MR = Matrix Reasoning (WAIS-IV); RK = Remember–Know; RLT (% correct post reversal) = Reversal Learning Task (percentage correct during reversal phase); RLT (switches after reversal) = Reversal Learning Task (switching responding after getting the first correct during reversal phase); SR = Scene Recognition; SCAP = Spatial Capacity Task; SMNM = Spatial Maintenance and Manipulation; SST = Stop-Signal Task; Stroop = Stroop Color-Word Task (mean reaction time on inhibitory trials); Stroop (interference score) = Stroop Color-Word Task interference (inhibitory reaction time—congruent reaction time); SP = Symbol Span (WMS-IV); TS = Task Switching (mean reaction time on switch trials); Task Switching (interference score) = Task Switching (postswitch reaction time—nonswitch reaction time); VCAP = Verbal Capacity Task; VF = Verbal Fluency; VMNM = Verbal Maintenance and Manipulation; VR = Visual Reproduction II (WMS-IV); Voc = Vocabulary (WAIS-IV).

The effect size of the group difference between community volunteers and patients (controlling for age and gender) for each cognitive test is shown in Figure 2. The largest difference between the groups consisted of reaction time variability on the Continuous Performance Task (η_p_^2^ = 0.044, *p* < .001), followed closely by total learning (Trials 1 to 5) on the CLVT-II (η_p_^2^ = 0.040, *p* < .001); these differences were in the medium range according to Cohen’s classification (Cohen, 1988). Of note, some of the cognitive test variables that had weak correlations with other tests (and thus not included in factor analyses) nonetheless showed small to medium effect sizes between groups, including signal detection (d’) on the Continuous Performance Task (η_p_^2^ = 0.024, *p* < .001) and SSRT from the Stop-Signal Task (η_p_^2^ = 0.021, *p* < .001). Interference scores on the Attention Network Test, Stroop, and Task Switching (in which reaction times for congruent stimuli were subtracted from incongruent stimuli) showed nonsignificant, minimal effect-size differences between the groups (η_p_^2^s < 0.01, *p* > .05), while the unadjusted mean reaction time of incongruent stimuli on these tests had significant effect sizes in at least the small range (η_p_^2^s > 0.018, *p* < .001). Finally, when factor scores derived from the factor analysis model were compared between the groups, the inhibitory control factor showed the largest group difference effect size (η_p_^2^ = 0.031, *p* < .001), followed by memory (η_p_^2^ = 0.018, *p* < .001). The verbal/working memory factor also showed a significant group difference (*p* = .017), although the effect size was small (η_p_^2^ = 0.006).

## Discussion

We examined the relationship between multiple experimental and traditional cognitive tests in a large community sample. This revealed that several experimental measures had weak relationships with all other tests and were inappropriate for factor analysis, including the Stop-Signal Task, Reversal Learning Task, Delay Discounting Task, Balloon Analogue Risk Task, and Spatial Maintenance and Manipulation Task. In the remaining tests that were included in factor analysis, a three-factor structure was supported, broadly consisting of tests that have been conjectured to measure verbal/working memory, inhibitory control, and memory, respectively. This three-factor solution was largely confirmed through MGCFA, in which the model was shown to have adequate fit in both community volunteers and patients relative to the number of factors, strength of factor loadings, and test-to-factor scaling, although error variance for the model was not equivalent between the groups (i.e., invariance uniqueness was not met). These findings supported the convergent validity of several experimental tests of working memory and memory.

The Verbal Capacity Task and the Verbal Maintenance and Manipulation Task were designed to measure verbal working memory by requiring the participant to hold letters in short-term memory or mentally manipulate (i.e., alphabetize) syllables, respectively. Consistent with this purpose, these tests loaded on a factor mostly composed of traditional tests considered to measure verbal and working memory, including Digit Span, Letter–Number Sequencing, Verbal Fluency, Vocabulary, Matrix Reasoning, and Color Trailmaking (listed in order of factor loading, respectively). It is encouraging that these results were found despite differences in the method of administration for tests loading within the same factor (i.e., by computer or with verbal stimuli administered by an examiner).

Two experimental tests of memory were included in the factor analysis, Remember–Know and Scene Recognition. These computerized tests evaluate recognition memory for words/drawings and photographs of locations, respectively. These tests loaded on a factor with other measures of memory and visual working memory, including Visual Reproduction, Symbol Span, CVLT-II, and the Spatial Capacity Task. The Spatial Capacity Task requires participants to maintain the spatial location of stimuli (i.e., dots) over brief intervals and is thought to be a test of visual working memory. Current results were consistent with this assumption, particularly given that it loaded together with Symbol Span, a subtest designed to measure visual working memory from the WMS-IV. Overall, this “memory” factor appears to reflect the ability to retain information over time (particularly visual information), whether or not that information needed to be mentally manipulated.

Two experimental measures of inhibitory control, the Attention Network Test (executive component) and Task Switching, loaded together on a factor with other timed measures of inhibitory control, including the Stroop Task. This broadly supports the view of these tests as measures of inhibition. However, these results were found when the dependent variable calculated from each test consisted of the mean reaction time on “incongruent” trials. In contrast, when interference scores were calculated from these tests, in which “congruent” reaction times were subtracted from “incongruent” reaction times, the interference scores had low correlations with all other tests, including each other. Unlike mean incongruent reaction time, interference scores also showed trivial, nonsignificant group differences when the scores were compared between the community volunteers and patients. These results call into question the common practice of creating interference scores to isolate inhibitory control from more general measures of processing speed. Prior research has shown poor test–retest reliability for interference scores, leading to the suggestion that they should not be used in the measurement of individual differences (Schuch et al., 2022; also see Heflin et al., 2011; Vendrell et al., 1995). Additional research is needed to determine the extent to which these tests can be considered “pure” measures of inhibitory control rather than measures of processing speed with an inhibitory component. Without additional support, we advise against using interference scores as primary measures of inhibition in clinical neuropsychological evaluations.

Because of weak relationships with all other cognitive test scores, some tests could not be included in factor analyses. These included the Stop-Signal Task, Reversal Learning Task, Delay Discounting, Balloon Analogue Risk Task, and Spatial Maintenance and Manipulation Task. These tests do not appear to measure aspects of verbal working memory, inhibitory control, or memory when these constructs are defined in the manner described herein, and based on the subject populations currently under investigation. This finding is perhaps not surprising for the Balloon Analogue Risk Task, which was the only test included in the CNP battery that intends to measure risky decision-making. In addition, delay discounting is not a performance-based test but rather a measure of preferences. Departing from prior research (Hinson et al., 2003), delay discounting was not related to measures of working memory (see also Shamosh et al., 2008).

Because the reliability of a test inherently constrains its validity, it is noteworthy that some of the measures that correlated poorly with all other tests have suboptimal reliability. The Balloon Analogue Risk Task, Delay Discounting, and reversal learning have modest to adequate reliability (Beck & Triplett, 2009; Waltmann et al., 2022; White et al., 2008), whereas test–retest reliability of the Stop-Signal Task is poor. Meta-analysis of stop-signal data from multiple studies demonstrated an aggregate test–retest reliability coefficient of only *r* = .34; the authors suggested that the task does not measure a stable trait (Thunberg et al., 2024). Interference scores from the Stroop and Attention Network Test likewise exhibit test–retest reliability coefficients below .35 (Jones et al., 2016; Schuch et al., 2022). To our knowledge, reliability of the Spatial Maintenance and Manipulation Task has not been evaluated.

Although the Stop-Signal Task did not show strong relationships with any other cognitive test, there was a small to medium effect-size difference between the community volunteers and patients on this test. This suggests that stop signal is sensitive to cognitive differences, broadly defined. It is possible that stop signal measures a state-like cognitive ability with unique functional correlates (not otherwise measured here), as has been shown for some other tasks developed in the cognitive neurosciences (see Cognitive Neuroscience Test Reliability and Clinical Applications for Schizophrenia [CNTRACS]; Gold et al., 2012). Conversely, particularly given poor reliability, stop signal may be sensitive to detecting differences at the group level but is not a precise measure at the individual level. Similar findings were shown for the switching of responses on the Reversal Learning Task during the reversal phase; scores were not highly correlated with other tests, but a small group difference was observed between the patient and community groups. Additional research on the correlates of these tests is needed for clarification.

Testing of measurement invariance suggested that the three-factor structure identified in the community volunteers was appropriate for application in the patients, although residual error of the model was not equivalent between groups. Equivalence of residuals is not a prerequisite for interpreting group differences in factor scores, and some researchers do not consider it a necessary step to data interpretation (Putnick & Bornstein, 2016; Widaman & Reise, 1997). Nonetheless, the finding suggests that unmodeled extraneous variables may have had differential effects between the groups. It is unclear what extraneous factors may be involved, but it is conceivable that psychiatric symptoms in the patients could produce differences in task engagement or testing approach that are not captured in the community-based model. These factors do not invalidate the model for patients per se but may lead to more measurement inaccuracy.

In sum, this study showed that some purported experimental measures of cognitive control had weak relationships with all other tests, while the convergent validity of experimental tests of working memory and memory was supported. Strengths of the study include independent validation of EFA results using MGCFA in separate samples, and diversity of the sample, in which tests were administered in both English and Spanish. Limitations involve the comparatively small size of the patient group (*n* = 137) and the circumscribed type of psychiatric diagnoses included (i.e., ADHD, schizophrenia, and bipolar disorder). The patients had not been evaluated for cognitive disorders, although cognitive deficits were undoubtedly present in some individuals. This limits the extent to which group differences in test scores between community volunteers and patients can be considered to reflect cognitive “impairment,” particularly as it relates to deficits in specific cognitive functions that may or may not have been present in the individuals tested. Comparisons between community volunteers and patients do, however, provide a general estimate of the sensitivity of the tests to detecting the type of cognitive differences that are known to be present in these psychiatric conditions (Fatouros-Bergman et al., 2014; Pievsky & McGrath, 2018; Robinson et al., 2006). Generalizability of current results can be extended by conducting analyses in samples with more severe cognitive deficits and/or focal deficits in particular areas of cognitive function. Evaluating validity of the tests through other means, such as through expert consensus (see Krause, 2012) and the ability of the tests to predict real-world functional problems (see Marcotte & Grant, 2010), is also strongly recommended.
